# Awareness increases acceptance and willingness to pay for low-carbon fuels amongst marine passengers

**DOI:** 10.1016/j.heliyon.2024.e24714

**Published:** 2024-01-23

**Authors:** Judit Nyári, Árpád I. Toldy, Mika Järvinen, Annukka Santasalo-Aarnio

**Affiliations:** Research Group of Energy Conversion and Systems, Department of Mechanical Engineering, Aalto University, FI-00076, Espoo, Finland

**Keywords:** Alternative marine fuel, Willingness to pay, Green shipping, Consumer preference, RoPax vessel, Public acceptance

## Abstract

One of the main applications discussed in decarbonising the marine sector is via alternative fuels, such as methanol and ammonia, produced from renewable hydrogen. These alternative, low-carbon fuels often come with increased prices and operational expenses for the vessel operators, which are ultimately reflected in the passengers' costs. Therefore, it is important to assess passengers' familiarity with expressions linked to decarbonisation and their willingness to pay this ‘green premium’ for alternative fuels. To assess these, we ran a survey-based study and collected close to 2000 answers through different channels from marine passengers, specifically from those travelling in the Northern European region on roll-on/roll-off passenger (RoPax) vessels. We found that most of the passengers prioritise environmental friendliness in marine fuels and are concerned about environmental issues. However, there seems to be a lack of knowledge about fuels and fuel technologies. Familiarity with certain alternative fuel-related expressions results in a more positive view of them. The observed willingness to pay is affected by the level of education, income, and place of residence, in addition to the level of concern about environmental issues, frequency of travel and spending on trips. Close to 80% of passengers are willing to increase their spending if the vessel is powered by a low-carbon, alternative fuel. As the results indicate that the more passengers know about alternative fuels and their benefits, the more willing they are to pay for them, it is recommended that RoPax operators invest in educating them.

## 1 Introduction

CO_2_ emissions from the maritime sector reached 1 056 million tonnes in 2018, which corresponds to 2.89% of global total anthropogenic CO_2_ emissions [1]. The International Maritime Organization (IMO) is responsible for regulations regarding international shipping, including those related to emission control and prevention of greenhouse gases (GHGs), NO_*x*_ and SO_*x*_. Accordingly, the IMO has set an ambitious target to reduce GHG emissions by 40% by 2030 and by 70% by 2050 compared to 2008 levels, which was further tightened in 2023 to reach 20% by 2030, 70% by 2040 and net-zero by 2050 [2]. Furthermore, the IMO urges that the uptake of low-carbon fuels should reach at least 5% by 2030 in international shipping. To achieve these targets, the IMO has set in place increasingly stringent regulations, especially in the so-called Emission Control Areas (ECAs) such as the Baltic Sea and the North Sea in Europe. NO_*x*_ and SO_*x*_ emissions already have tight limits, e.g. the sulfur content of fuel oil is maximised at 0.5% on international waters, and 0.1% for vessels operating in ECAs [3], [4].

Furthermore, the European Union has its own targets for emission reduction within the maritime sector, while the inclusion of the sector in the Emission Trading System (ETS) has been under consideration for some time and was accepted in 2023. According to the accepted revision ETS [5], the maritime sector is included from the start of 2024, with an initially limited but gradually expanding scope of vessel types and sizes, considered emission levels and types of GHGs. The available cap will be reduced each year, and if an operator cannot meet the required amount of allowances, they have to pay 100 for each tonne of CO_2_ above their allowance in addition to surrendering the allowances. The EU has presented the FuelEU Maritime proposal [6] within its “Fit for 55” package. The proposed regulation introduced increasingly stricter limits on the carbon intensity of the energy used by commercial vessels of 5000 gross tonnage and above, regardless of their flag from 2025 onwards [7]. It covers all energy used on board when the ship is at and on voyages between EU ports, while for voyages departing from or arriving at an EU port 50% of the used energy is covered. According to the proposal, the carbon intensity would decrease at an accelerating annual rate, ultimately resulting in a reduction of 75% by 2050 compared to the 2020 base year. Full life-cycle GHG emissions must be taken into account, not just emissions directly originating from fuel usage by the vessels. However, the proposed regulation has been criticised [8] because it would enable the use of fossil-based liquefied natural gas (LNG). LNG has low emissions during use and would therefore fulfil the criteria, even though it has high production emissions and leakages within the transmission system [9]. Instead, a counter-proposal urged that sub-targets for renewable- and biofuels should be added to the regulation. Also, they proposed higher cuts to GHG intensity from 2035 onward – 20% as of 2035, 38% from 2040, 64% as of 2045 and 80% as of 2050. More recently, a target of 2% for the use of renewable fuels of non-biological origin from 2030 was proposed to be added to the FuelEU Maritime proposal [10]. The EU bodies have listened to this criticism and reached an agreement in 2023 [11], [5]. The regulation is currently awaiting formal acceptance from the European Parliament and the Council and will enter into force from 1^st^ January 2025. The regulation describes the required reductions as 2% by 2025, 6% by 2030, 14.5% by 2035, 31% by 2040, 62% by 2045, and finally 80% by 2050 from the starting GHG intensity value of 91.16 g COe2/MJ. Furthermore, a sub-target was added for renewable fuels of non-biological origin, and compulsory connection to on-shore electricity supply at ports for container and passenger vessels from 2030 onwards.

Transportation of goods and passengers by sea is a common and growing European market [12]. In this research, the focus is solely on RoPax vessels, defined as a specific type of sea vessel that combines the transportation of goods via roll-on/roll-off features for commercial vehicles with passenger carriers equipped with cabins and entertainment services. Until recently, these vessels have solely used regular maritime gas oil (MGO) or heavy fuel oil (HFO), two of the most polluting fuels accessible [1]. CO_2_ emissions within this segment globally reached 36.7 million tonnes of CO_2_ [1], out of which approximately 40% originated from the European Union [13]. After a significant drop both in passenger and CO_2_ emission levels due to the COVID-19 pandemic, these numbers are back to pre-COVID levels [12].

There are several methods to mitigate emissions from the shipping industry, such as advanced logistics and digitalisation, improved hydrodynamics, more efficient machinery, post-treatment of flue gases, and low-carbon fuels [14], [15]. Amongst the solutions, a report by DNV [14] concludes that low-carbon fuels could achieve the most significant emission reductions. However, an IMO report [1] states that while the CO_2_ abatement potential of these fuels is significant, their cost is also one of the highest amongst the investigated technologies [16]. These low-carbon or alternative fuels, e.g. LNG, methanol, and hydrogen, are the sum of fuels that either through production or utilisation, but especially during their full life-cycle, emit significantly lower amounts of CO_2_, especially compared to HFO and MGO [14]. According to DNV's report [14], currently, 1.2% of all globally operating vessels use some kind of alternative fuel. However, it is promising that 21% of all ships on order are equipped with engines that are capable of running on alternative fuels. According to DNV's estimations, by 2050, a fuel mix of methanol, ammonia, and hydrogen will be produced from biomass or renewable electricity. In addition to this, fossil fuels will remain in use, but their emissions will be mitigated using onshore and onboard carbon capture technologies [14].

Alternative fuels are generally more expensive than HFO and MGO, especially when synthetic fuels produced from green hydrogen are compared to their fossil-based counterparts [16]. To compensate for their increased cost, RoPax operators might increase their prices, as has happened during 2022 [17]. Therefore, it is important to assess the amount customers would be willing to pay for these alternative fuels. Most studies measuring consumers' willingness to pay (WTP) within renewable energy-related topics are either carried out about electricity [18], [19], [20] or passenger vehicles and fuels [21], [22], [23]. The studied literature agrees that the more a consumer knows about green and renewable technologies, the more they are willing to pay for them [20], [21], [19]. Hackbarth and Madlener [22] also states that there are above-average interested groups that should be specifically targeted for alternative fuel vehicle possibilities. Li and McCluskey [23] has shown that average respondents were willing to pay 11% more for a second-generation biofuel than fossil fuel. They also agree that environmentally conscious, well-informed respondents were willing to pay even more above the average. Furthermore, willingness to pay for renewable energy increases when it is framed with a positive image and when it is highlighted that the consumers' choice has a significant effect on the future [18].

The RoPax sector started investments into alternative fuels as early as 2015 with the operation of Stena Line's Germanica running on methanol [24], and since 2017 Rederi AB Gotland's vessel running on LNG as the main fuel [25]. Since then, the segment has continued to be the front-runner of decarbonisation efforts in the maritime sector [12]. Moreover, there are significant investments at regional ports in Northern Europe to create hubs for these alternative fuels [26], [27].

While there have been recent studies conducted in the maritime sector about the WTP of a product shipped by LNG-fuelled vessel [28], and marine customers [29], according to the authors' knowledge, no similar study has been carried out about the passengers in the maritime sector. This research collected data from RoPax passengers travelling in the Northern European region. Here, the Northern European region is defined as the Gulf of Finland, the Gulf of Bothnia, the Baltic Sea, the North Sea, the English Channel, the Irish Sea, and the Norwegian Sea. This research observes the willingness of people to pay for a low-carbon fuel as an increase in the ticket price or to compensate for their CO_2_ emissions voluntarily. Besides the stated WTP observations, it also assesses the passengers' preferences and knowledge about different fuels and climate change mitigation tools in the maritime sector. The article consists of four sections after the Introduction. First, the current status of alternative fuels is described to provide an overview and to provide background information on the analysis of the passengers' responses. Then, the Methodology is described, where the survey design, data collection, sampling and analysis are shown in detail. This is followed by the Results and discussion, and the Limitations and future work sections, and finally, the Conclusions are provided.

## 2 Background of marine fuels and their production methods

Maritime transportation, especially on long distances, is considered one of the hard-to-abate sectors due to the difficulty of direct electrification [30]. While short-distance and coastal shipping can utilise batteries, and it is the most ecological solution [31], [32], this solution is not yet available for long-distance shipping due to the size of the battery and the frequency of charging [30], even though some researchers propose that battery-electric ferries will be more financially competitive by 2030 than internal combustion engines [33]. While hydrogen is often discussed as an upcoming fuel in the marine sector due to being nearly emission-free when produced from renewable electricity, in the near future, hydrogen-fueled vessels will only be available for coastal and short distances [34]. For long-distance maritime transportation in the near future, alternative fuels and emission mitigation technologies are necessary to reduce emission levels [35]. Furthermore, to cut overall emissions, combustion and production emissions need to be reduced [36]. Yet, at the same time, the fuels need to fulfil certain characteristics, such as energy density and compatibility with current infrastructure, with minimal investments into retrofitting. For example, it is not worth switching to a fuel with significantly lower energy density at the expense of cargo capacity. Furthermore, the safety of crew, cargo and passengers must be considered, as well as the effect of a potential leak into the marine ecosystem. To achieve net-zero emissions from any sector, not only are fuels needed that emit less while being used, such as direct electrification or hydrogen but also their production method needs to shift from fossil feedstocks to biomass-, waste- or non-fossil electricity-based alternatives. Chemically and physically, the fuels made from traditional fossil feedstocks and alternative feedstocks can be identical, and their emissions are the same during utilisation.

Due to new regulations for sulphur emissions, low sulphur fuel oils (LSFOs) such as marine gas oil (MGO), marine diesel oil (MDO), very low-sulphur fuel oil (0.5% m/m sulphur content), and ultra-low sulphur fuel oil (0.1% m/m sulphur content) have emerged [37]. These are based on HFO either as distillates or as blends. HFO, MDO, and MGO are considered the current traditional fuels for maritime transportation [14] due to their low cost and availability [38]. LNG is the most widespread of the alternative fuels used for maritime transport due to its low cost, availability, high energy density and compatibility both technically and to IMO regulations [14]. While LNG - as its name indicates - is made from natural gas, it can also be produced by biogas upgrading to liquified biogas (LBG) or liquified biomethane (LBM) [39] or from renewable hydrogen and CO_2_ [14] to obtain synthetic LNG (SNG). The main setback of using LNG, LBG, LBM or SNG is their gaseous state under standard conditions, which necessitates investments in compression equipment. Furthermore, a potential methane slip or system leakage could diminish any of the environmental benefits that LNG offers as methane has a significantly higher global warming potential than CO_2_ [40], [41], [9].

Methanol has been discussed as one of the most promising fuels for the shipping sector in the near future. There are already vessels that use either solely methanol or methanol-diesel dual-fuel combustion engines [42]. Furthermore, methanol can be used in fuel cells as well, at the moment, vessels are being built that use fuel cells at the port and not as the main power source [43]. Because methanol is liquid at standard conditions, no further compression is needed to store it. However, its energy density is lower than that of regular fuels such as MGO (Table 1), which means it occupies more storage space from the valuable cargo. Methanol combustion emits CO_2_, while there are no SO_*x*_ emissions, low particulate matter emissions, and lower NO_*x*_ emissions compared to MGO [44]. Traditionally, methanol is made from natural gas, but it can also be produced from biomass or as a synthetic fuel from hydrogen, and CO_2_ [45]. Inhalation of methanol gas, as it is denser than air, is highly toxic, while its ingestion can lead to death, similar to other fuels. However, methanol is biodegradable and water-miscible, which means that even in large quantities, it is not harmful to the environment [46].

**Table 1:** Comparison of alternative fuels for maritime transport based on Zou and Yang [57].

	HFO	MGO	LNG	Methanol	Ammonia
Lower heating value (MJ/kg)	40.85	42.8	47.5	21.4	18.7
Energy density (MJ/m^3^)	39120	35800	21410	17850	12717
Storage medium	Liquid	Liquid	Cryogenic liquid	Liquid	Compressed liquid
Storage pressure (bar)	1	1	1	1	10.3
Storage temperature (C)	25	25	-162	25	25

Ammonia (NH_3_) has been considered due to being carbon-free and sulphur-free. Therefore, it does not emit CO_2_ or SO_*x*_ during combustion [47]. However, although several studies focus on the bottlenecks of ammonia supply and production [48], the major drawback of using ammonia is the nonexistence of engines capable of running on it. Even though extensive research, currently, only dual ammonia-diesel engines exist [49]. Furthermore, ammonia can be used in fuel cells as well, similarly to methanol, with several ongoing initiatives for the marine sector [50]. As ammonia is a gas at standard conditions, it is stored under pressure in liquid form, which is an energy and cost-intensive process. It is toxic when in contact with the skin or inhaled, and as a gas, it can escape more easily during a leakage. For the aquatic environment, ammonia is a direct toxin as it results in increased pH and temperature levels [51]. It can be made sustainably from renewable hydrogen and captured nitrogen through the Haber-Bosch process, similar to fossil natural gas-based ammonia [52]. Emissions from ammonia are NO_*x*_ and N_2_O at significant levels, the reduction of these requires further technical development [53].

Other biofuels such as hydrogenated vegetable oil (HVO), and fatty acid methyl ester (FAME), both often referred to as biodiesel, also provide significant emission reduction benefits compared to MGO, especially when produced from waste streams instead of virgin biomass [54]. HVO and FAME are recommended to be used as drop-in solutions for blending with existing fuels [55].

A summary of different alternative fuels can be found in Table 1. Hansson et al. [34] also compared some of these amongst other fuels, which involved expert opinions from Swedish maritime stakeholders. In their paper, it was evident that vessel operators, engine and fuel suppliers have different criteria than government authorities. While the former prioritised economic and technical criteria, the latter preferred environmental and social criteria. Overall, it can be said that there is not a single specific fuel that would be perfect, but instead, a mixture of production methods and a proper fuel mix can enable net-zero emissions for the long-term future goals of the shipping industry [14]. As can be seen from the summary provided here, there is no “silver bullet” on the market, each fuel has its limitations, challenges, advantages and disadvantages [56].

## 3 Methodology

In this section, the detailed questionnaire design is described. This is followed by the data collection method and the sampling of targeted respondents. Finally, data analysis methods are elaborated.

### 3.1 Survey design

The questionnaire consists of four parts: 1) background information of the passenger, 2) travelling habits and preferences, 3) climate change mitigation and fuels, and 4) willingness to pay. The design of the questionnaire is based on the works of Moula et al. [21] and Sonnenschein and Mundaca [58]. The questionnaire was available only online, where first, the respondent could select between different languages. It was translated into nine languages from the original English and was available in Danish, Dutch, Estonian, Finnish, German, Norwegian, Polish, Russian, and Swedish. The developed questionnaire can be found in English in Appendix 1.

In the first part, general demographic background information is collected from the respondents after a short introduction to the research and its purpose. These are age group, gender, education level, occupation, size of household, monthly net income of the household, and country of residence. Each question was made compulsory, but the respondents could select not to answer the questions. The monthly net income was exchanged to the local currencies of countries to whose language the survey was translated and are not in the euro-zone, i.e. Danish krone for Denmark, Norwegian krone for Norway, Polish zloty for Poland, Russian ruble for Russia, and Swedish krone for Sweden.

The second part assessed the travelling frequency and preferences when selecting between operators. In this part, three questions were asked. First, the frequency of travelling in the Northern European region was asked, along with a map to provide a better understanding of the interested region. Second, we asked the respondents to choose a maximum of three factors without an order of preference about their priorities when selecting between RoPax operators. We have presented the respondents with ten different options and also made them available to add their own selection criteria. Finally, in this part, we asked the respondents to estimate their per-person spending for a trip, including vehicle transportation but not including meals and other shopping.

In the third part of the survey, we asked the respondents about their environmental concerns, knowledge, and preferences regarding fuels in the maritime sector, especially different alternative fuels, and their production methods. While passengers are not experts in these topics, our intention was to understand what they consider important features and what their current knowledge and perception are about fuels and fuel technologies. We have provided only short examples of feedstocks to describe the different fuel production methods in questions 15 and 16 because we wanted the respondents to understand these methods the same way. We defined traditional fossil-based production methods as ones where the feedstock is e.g. coal, crude oil or natural gas, while biomass-based production was given by corn and sugarcane. Renewable production was given by waste, residue and algae feedstocks, and finally, synthetic production from renewable electricity. The use of these examples instead of precise definitions (such as first, second, etc. generation biofuels, or a full elaboration of power-to-X processes) both facilitates common understanding among the respondents and helps gauge their familiarity with the given terms. Furthermore, we asked them to select their preference from a list of climate mitigation tools and to choose a responsible group or institution that should, in their opinion, deliver CO_2_ abatement in the sector.

In the final part of the questionnaire, there were two main questions about the respondents' willingness to pay. First, we asked whether the passenger would be willing to compensate for their CO_2_ emissions voluntarily. For negative answers, the questionnaire asked the respondent to provide a reason available from a list. For positive answers, the respondent could choose between two options: to determine this compensation, either a fixed price or a by-distance-travelled model could be chosen. For both choices, respondents were prompted to select from a list of maximum values they would be willing to pay. Finally, a similar but more specific question was asked in the questionnaire. Respondents could choose the additional amount they would be willing to pay for a low-carbon fuel used for their trip. A short definition for low-carbon fuels was provided so that the respondents would have a clear and identical understanding of the expression. Again, a rejection of paying extra for such fuels was followed by a question to provide a reason for the selection. Finally, the questionnaire ended with a voluntary comment section.

### 3.2 Data collection and sampling

The online questionnaire received answers between the 22^nd^ of September and the 11^th^ of November 2022. The questionnaire was circulated in multiple formats, specifically targeting the customers of RoPax operators. Firstly, eleven RoPax companies operating in the given region were contacted to cooperate in the research. These companies have a cumulative passenger number above 30 million annually [59], [60], [61], [62], [63], [64], [65], [66], [67], [68]. Employees of RoPax companies were contacted and presented with the research problem and were asked for permission to conduct the study on their premises or online through their own social media. We asked them to share the link to our survey on their social media platform, in their newsletter, on board, or in their terminals with a poster including a QR code to the survey. We also offered to conduct the research on their premises at the terminals or onboard in person. Out of the contacted companies, three were willing to participate in sharing the survey with their passengers or allowing us to conduct the research in person. The three participating companies' number of passengers is 17.6 million annually, which is close to 60% of the approached RoPax operators' annual value. Moreover, different social media groups were targeted where travelling by RoPax vessels was the main topic of the group. We intended to target both international and regional groups and RoPax operator-specific groups. However, these groups were unavailable or did not exist in all the regions, therefore, data from certain countries can be minimal even though ports and companies are operating in the region. These groups were messaged with a short introduction to the research and its purpose, followed by a link to the survey.

All the data was collected anonymously and on a voluntary basis, both when collected on the field and through newsletters and social media posts. In the final data, there is no distinction between the answers based on how the data was collected, as data regarding that was not collected.

### 3.3 Data analysis

The survey was developed in Webropol, which is a freely available professional data management tool at Aalto University. The software provides text mining, analytics and simulation of data above the design of surveys and collection. For most of the questions, descriptive statistical methods were used for analysis. Regarding WTP observations, t-statistical tests and analysis of variance (ANOVA) tests were calculated for two-variable questions and questions with more than two variables, respectively. Some of the answers to questions with more than two options were grouped. T-tests and ANOVA were used as even though WTP has a non-normal distribution [58], the sample size in this research was expected to be high. Moreover, the effect size measures were added because, at a high sample size, the difference between groups can be statistically significant, but that does not mean that this difference is meaningful. Cohen's d value was calculated for t-tests, and eta squared was calculated for ANOVA to understand these differences. For each t-test and ANOVA, the null hypothesis was that there was no difference in the means of the groups and for all tests, the level of significance was p = 0.05.

## 4 Results and discussion

During the seven weeks when the survey was available online, close to 4000 people opened the survey (each opening counts as an individual one even from the same IP address), and close to 3000 people have started to respond to the survey. Finally, there were 1914 submissions of the survey that are analysed in this section. Considering those who started to respond to the survey as 100%, the response rate was 64%, which is significantly higher than the average at 44% measured by Wu et al. [69].

As described earlier, there is no way of knowing in the final data whether the answer was collected through a newsletter, social media groups or in person on the field. However, it can be said that collecting data through online media generated more openings for the survey but fewer finished surveys. Meanwhile, in-person data collection resulted in more finished surveys after having opened the link. Moreover, collecting data online was more cost and time-effective compared to in-person travelling to destinations. We consider the collected data as a representative sample of RoPax passengers in the Northern European region, and we also assume that the given answers are truthful.

### 4.1 Passengers' demographics and market preferences

Background information was collected about the passengers, including their gender, age, education, type of employment, size and total net income of the household and country of residence. The main findings can be found in Table 2. Two-thirds of the respondents were male, which could be attributed to the interest of the social media groups that focus on sea transportation and ship spotting. Furthermore, close to 75% of the respondents were above the age of 55. This is explained by that this type of travelling method is more frequent among pensioners as they have more time to use more time-consuming transportation methods, and they are more likely to read newsletters [70], [71]. Almost 70% of the respondents had at least completed high school studies, while 43% had some level of higher education degree. Corresponding to the age distribution, almost half of the respondents were pensioners, while around 45% worked in some way, and the participation of students and unemployed respondents was minimal. Most of the respondents lived in a two-person household, while 20% alone, and one-third lived with at least two other people. Income level was the question that most of the respondents, more than 20%, refused to answer. The monthly median income was between 2501 and 4000  per household, one-fourth of the respondents belonged to this group, otherwise, the distribution showed normal distribution. Regarding the country of residence of the respondents, it is clear that responses from some of the countries are over-represented in the data while others are under-represented. The main reason behind this is the newsletter that was sent out by one of the approached RoPax operators. Also, the social media groups were not covering the whole region. Therefore, responses from the countries where this RoPax operator has its routes within the Northern European region are over-represented.

**Table 2:** Socio-demographics of respondents.

Parameter		Value	Unit
Total number of respondents		1914	
Share of women		33	%
Age (median)		55-74	years
Size of household (median)		2	persons
Net income of household (median)		2501-4000	/month
Share of pensioners		48.6	%
Share of higher education		68.4	%
Country of residence	Great Britain	44.5	%
	Norway	13	%
	Denmark	14.7	%
	The Netherlands	12.6	%

Most respondents travel by RoPax once or twice annually, however, 10% of the respondents travel more than 5 times. 74% of the respondents travel at least once and therefore can be called regular passengers of RoPax vessels, while 32% travel more than twice annually. The most important factors in order when selecting between operators were price, the RoPax route and finally, the location of ports and terminals. This means that respondents primarily look for specific routes, yet, in the case of similar routes, the respondents are increasingly price-sensitive. Loyalty programs and services provided at the destination were the least important factors, according to the respondents. More than 50% of the respondents spend at least 150 on their trip, and 20% of them spend more than 300 per trip. Meanwhile, 15% of the respondents manage their trips below 50.

### 4.2 Passengers' view on climate change mitigation and fuel preferences

Most of the respondents, more than 80%, are at least somewhat worried about environmental issues, giving at least 5 on the 0-10 scale (where 0 meant “Not worried at all”). Almost 45% are seriously worried about these issues and gave at least 8 on the scale. Respondents considered global warming as the single most important environmental concern. It is followed by five other similarly worrisome issues, such as air and water pollution, overpopulation, deforestation and natural resource depletion.

**Figure 1:**
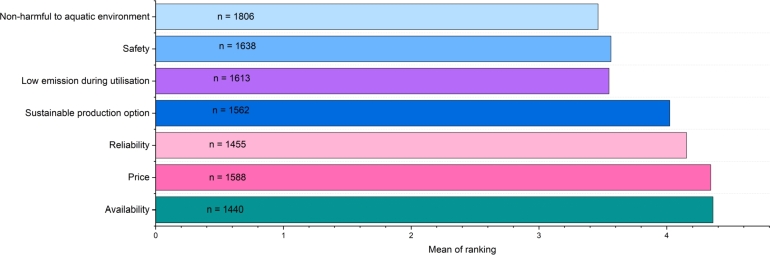
Mean of ranking of fuel characteristics, where means 1 means ranked first out of seven, and *n* means the total number of selections regardless of ranking.

When asked to rank the seven given characteristics of marine fuels according to importance, non-harmfulness to the aquatic environment was selected the most often, and it was also the most important characteristic. As it was not compulsory to select and rank all the listed characteristics, there is a difference in the number of selections for each of them, as shown in Fig. 1. It was compulsory to select three out of the seven given features, still, more than 60% ranked all seven of them. Overall, the second most important feature of marine fuel, according to the respondents, is to have low emissions during utilisation. In the third place, safety was selected, while the fourth was the possibility of producing the fuel in a sustainable manner. It is clear that out of the listed characteristics, overall environmental friendliness and low emissions are the most vital to the respondents. Even though environmental factors were not important for respondents when selecting between RoPax operators, and the most important factor was pricing, an opposite pattern is seen when specifically considering fuels used in RoPax vessels. Price was selected as the second least relevant characteristic of marine fuel. This is also interesting in light of 2022's energy crisis in the EU, as a sudden decrease in the availability of fossil fuels has led to increased prices in all sectors.

When passengers were asked to select the most suitable fuels for RoPax vessels, more than 40% of them could not select any of the listed options. This might mean that passengers, in general, have inadequate knowledge about fuels used in maritime applications, and they have not even tried to pick one, opting to honestly admit that they cannot tell which of the given options would be the best. The 60% who have selected a fuel from the list chose hydrogen as the most suitable option for RoPax operations. One-third of those who selected a fuel think that hydrogen is the most applicable fuel, followed by direct electrification, selected by more than 10% of all respondents. Ammonia and methanol were deemed appropriate fuels by less than 2%, even though, as shown in Section 2, these are considered to be the top candidates for future marine fuels.

**Figure 2:**
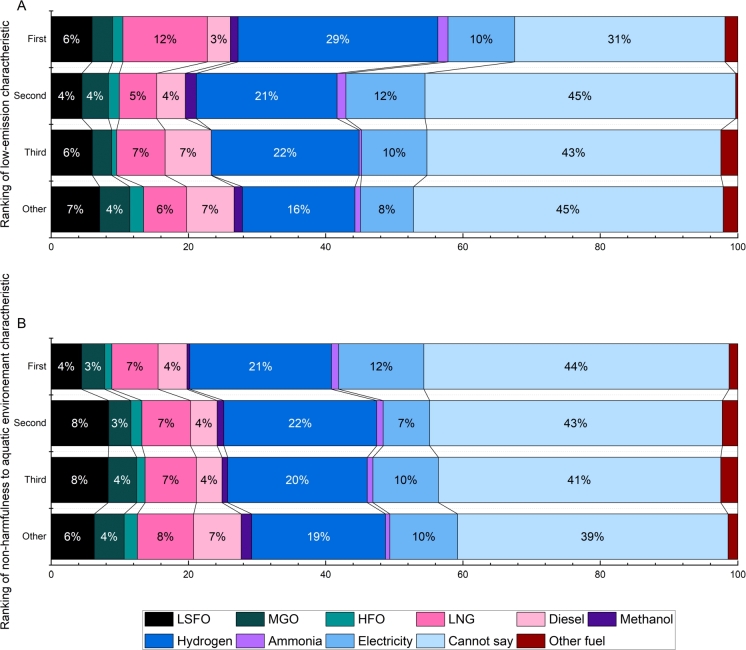
Selection of most suitable fuel versus the ranking of low-emission characteristic (top) and non-harmfulness to the aquatic environment (bottom).

Selecting hydrogen and electricity goes in line with the features marked important by the respondents, as both of these energy sources are non-harmful to the aquatic environment and can be produced from sustainable sources. It was expected that the selection of the most suitable fuel would agree with the importance of its characteristics. Therefore, those who selected low emissions during utilisation as an essential aspect chose fuels with lower emissions. Similarly to non-harmfulness to the aquatic environment, those who ranked it as a more important feature of the fuel selected fuels that fulfil this criterion than those to whom this feature was less important. According to Fig. 2 (A), those who considered low emission during utilisation as the most important feature could decide between the fuels and primarily chose hydrogen. There is an overwhelming popularity for hydrogen regardless of how respondents indicated their preferences for fuel characteristics. LNG and electricity are the other two preferred fuels for those considering low emissions as an essential criterion for marine fuels. Regarding the correlation between non-harmfulness to the aquatic environment and the most suitable fuel, see Fig. 2 (B), again hydrogen was the most popular choice, as around 20% chose it regardless of the ranking of the given feature. It seems those who considered low emissions the more important feature selected a fuel with higher certainty than those who chose non-harmfulness. There are respondents who, while believing that these criteria are essential, cannot select a fuel which would meet them. It is also clear that alternative fuels that receive less publicity in mass media but can nevertheless fulfil these criteria better than LSFO or diesel are less likely to be selected (such fuels include ammonia and methanol).

When passengers were asked to select how the fuel should be produced, most of them, almost 40%, selected that a combination of production methods would be the best solution. Overall, renewable sources-based production of fuels, here defined as production from wastes, residues and algae, was the most favoured. 16% had no preference at all for how the fuel was made, while the least selected method was traditional fossil-based production. The most popular combination of production methods was renewable & synthetic production. It is somewhat surprising that biomass, when described as a first-generation feedstock, was not a popular selection on its own (only 6% selected it), while in combination with renewables, it was significantly more favoured. In summary, renewable production on its own was selected the most, by more than 21%, second was renewable & synthetic with 19%, and in third place, both synthetic production on its own and biomass combined with renewable production were selected by 12-12%.

**Figure 3:**
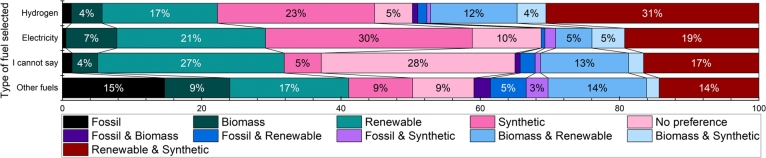
Selection of most suitable fuel and its production method, here “Other fuels” is every other answer than hydrogen, electricity and “I cannot say”.

Fig. 3 shows how selecting the most preferable fuel compares to its production method. The majority of those who could not select a fuel marked no preference mostly for the production method, which is evidence that they have the least knowledge about fuels and their production methods. However, one-third of them selected renewable, waste-based production, and a bit over 12% chose renewable & synthetic production. The majority of those who could select between the different fuels also had a preference for its production. More than one-third of those who selected hydrogen as the most suitable fuel chose renewable & synthetic production methods for it, while almost a fourth selected solely synthetic production. For electricity, the most preferred production method was renewable electricity, chosen by one-third. As described above, renewable electricity was represented by the term “synthetic”, while “renewable production” referred to waste and residue-based methods in the questionnaire. Renewable production and renewable & synthetic production was picked by around 20-20% of those who preferred electricity. Those who selected fuels other than electricity or hydrogen had less clear preferences towards production methods, as four of the methods were picked by 15-15%: renewable, fossil, renewable & synthetic and biomass & renewable. It is evident that in all groups, synthetic, renewable, and their combination were the most dominantly selected production methods, while fossil and first-generation biomass-based productions were less preferred.

**Figure 4:**
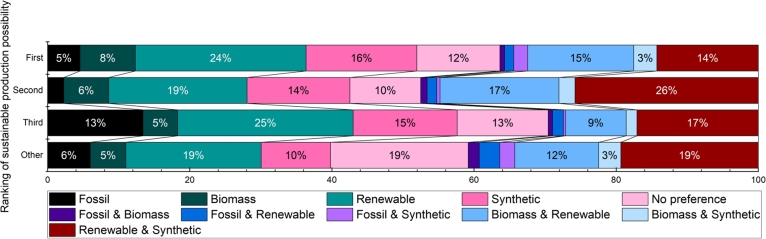
Selection of fuel production method and the ranking of sustainable production method possibility.

Those who have marked the possibility of the sustainable production method as high importance versus how they selected the production method can be seen in Fig. 4. Regardless of the importance of sustainable production methods in the ranking, the respondents selected renewable or renewable+synthetic production methods primarily. These were followed by synthetic and synthetic+bio-production methods. The only divergence from this is the group that ranked sustainable production as third, where fossil production is the fourth preferred method. 20% of those who ranked sustainable production less than third indicated that they do not have a preference for the production method, while another 20-20% chose either renewable or renewable+synthetic production methods.

When passengers were asked about their knowledge of specific terms, the expression known most widely by respondents was “fossil fuel”, known by 79%, while the second best-known term was “biofuel”. 8% of the respondents claim that they are not familiar with any of the listed names, which is a surprisingly high value. However, as stated earlier, we consider all the answers sincere. The expressions “synthetic fuel”, “low-carbon fuel”, “green fuel”, and “renewable fuel” are known to 35-40% of the respondents. The expressions “electrofuel”, “e-fuel”, “powerfuel”, and “sustainable marine fuel” are known by 11-21% of the respondents, while “power-to-X”, “power-to-liquids”, “alternative marine fuel”, and “emissions-to-liquids” are known by less than 10%.

**Figure 5:**
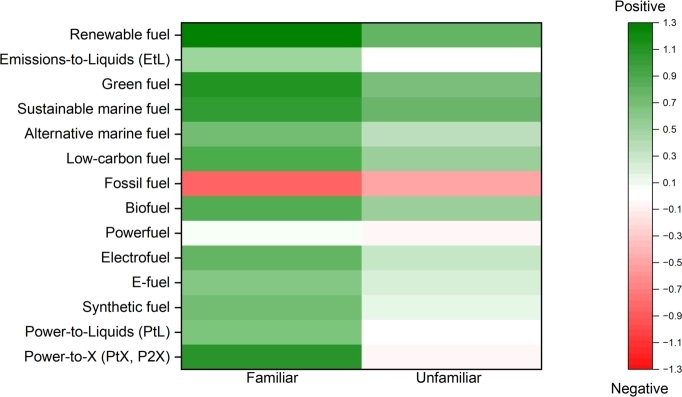
Comparison of perception of fuel-related expressions by those who marked them familiar versus unfamiliar.

We then compared whether there is a difference in how different fuel-related expressions are perceived amongst those who are familiar with it versus those who are not, see Fig. 5. It is obvious that those who are familiar with the expressions have stronger perceptions, either more towards the positive or the negative end, while those who are not familiar with the given expression are more likely to choose “neutral”. Out of the top five positively viewed expressions, four are the same for both of the groups, and these were in no specific order: low-carbon fuel, sustainable marine fuel, green fuel and renewable fuel. The fifth expression for the group which was familiar with the expression was power-to-X, which was a negative expression for those who were not familiar with it, while those who were unfamiliar with biofuels marked it in their top five positively perceived expressions. The most negative connotation was towards “fossil fuel” in both of the groups, and for the familiar group, it was the only expression that had a negative perception.

We also compared the perceptions of fuel-related terminology for languages where the respondents' amount was above 200, i.e. English, Dutch, Norwegian and Danish, and whether there is a difference between them. English and Dutch respondents found five expressions that were positive to them which were biofuel, sustainable marine fuel, green fuel and renewable fuel for both groups, in addition to electrofuels for the Dutch speakers and low-carbon fuel for the English speakers. In Danish only biofuel, sustainable marine fuel and green fuel sound positive, while for Norwegians, these were only biofuel and renewable fuel. At the same time, Norwegians found most fuels towards the negative end, including PtX, powerfuels, fossil fuels, and emissions-to-liquids. Fossil fuels were the most negative expression in all languages, followed by PtX in English and powerfuels in Dutch.

Somewhat over one-third of the respondents think that the RoPax operators should be mainly responsible for decreasing CO_2_ emissions. The rest was divided between the national governments, fuel suppliers, the EU and international non-governmental organisations such as the IMO. The least selected option was the passengers. However, several respondents commented that all parties should step up to bear their share of responsibility. Half of the respondents would decrease the emissions by opting for a low-carbon alternative fuel. One-third believe that engine improvements would be the best solution to achieve the reductions. A minimal number of respondents selected other options.

### 4.3 Passengers' willingness-to-pay for CO_2_ emission compensation and low-carbon marine fuels

**Figure 6:**
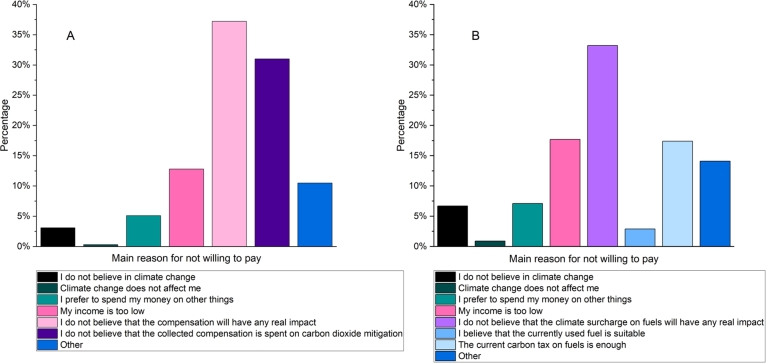
Main reasons indicated by respondents for not paying for voluntary compensation (left) and low-carbon fuels (right).

**Figure 7:**
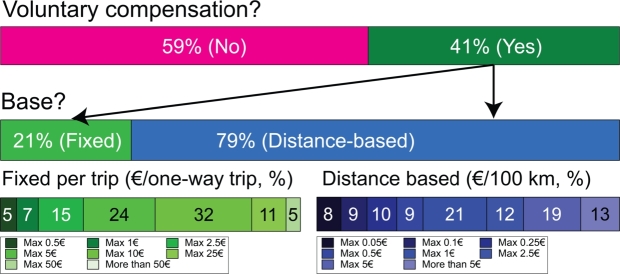
Share of voluntary compensation, their base of calculation and the amount passengers would be willing to spend per one-way trip or distance.

Close to half, 41% of respondents, would be willing to pay to offset their CO_2_ emissions voluntarily, as can be seen from Fig. 7. Those who would not pay reasoned that either the compensation would not have any real impact (37%), or they do not believe that the collected compensation would be spent on carbon dioxide mitigation (31%) as shown in Fig. 6(A). The majority, 79% of those who would be willing to compensate for their emissions voluntarily, would pay it proportionally to the length of the trip. Around 85% of them would pay at least 1  per one-way trip voluntarily, and almost half would be willing to pay at least 5  per one-way. Only 5% would pay a minimum of 25  per one way, however, none of them would pay more than 50 . Of those who selected the proportional payment, 75% would pay at least 0.25 per 100 km, while 45% would be willing to pay as much as 1 for this distance. One-third would even pay as high as 2.5, and 13% is willing to pay above 5 per 100 km to compensate for their CO_2_ emissions. This latter figure means that for a Helsinki-Stockholm one-way route, the price would increase by 22, while for a Helsinki-Rostock trip, this equals an additional 65. Finally, those who were willing to pay voluntary compensation were asked how they would prefer it to be used. 60% of them would invest it in carbon dioxide mitigation equipment, while one-fourth would buy low-carbon fuel. The rest of the options received less than 5% of the responses.

Observations about willingness to pay (WTP) for low-carbon fuel are presented last. One-fifth, 22% of the respondents, would not pay more, and they justified this by not believing that the climate surcharge of fuels would have any real impact (33%), while 18% reasoned that their income is too low, and 17% think that the current tax on fuels is enough and no further taxes and surcharges should be collected, see Fig. 6 (B). Overall, close to 80% of the respondents stated that they would be willing to bear an increased fare price if the vessel ran on a low-carbon fuel. Amongst them, two-thirds would pay at least 1 more per one-way trip, which is a rather small amount considering that half of the respondents spend at least 150 on their trips. Close to half, 44% would pay a minimum of 5, while less than 10% would go higher than 25. One-fifth of the respondents indicated that they would pay neither voluntarily nor for low-carbon fuel, and the main reason behind this is that they do not believe that any of these payments will have a real impact.

To further understand what influences the WTP of passengers, t-tests and ANOVA were conducted for several background questions. Table 3, Table 4 contain the results of these statistical tests. Regarding age, gender, occupation and size of household, it can be said that there is no statistical difference among the different groups. Education, income and place of residence, however, are statistically significant factors influencing WTP selection. Education and income both positively affect WTP. While education and place of residence have a small effect size, income has a negligible effect [72]. The frequency of travelling and spending is also statistically significant. However, while less frequent travellers are willing to pay more for low-carbon fuels, those who spend more on the voyage itself would also pay more for the low-carbon fuel. Out of these two factors, spending has a larger effect than frequency, although it is also small. Out of all the investigated factors, worrying about environmental concerns has the largest effect on passengers' WTP. The more a passenger is concerned, the more they are willing to spend. Other statistically significant correlations with WTP are mostly connected to other environmentally conscious choices. Those who selected other than fossil-based fuel production methods, those who selected low-carbon fuel as their preferred marine fuel and those who selected low-carbon fuel as the emission mitigation method have all higher WTP than those who selected anything else, and the effect size is small for all three cases. Interestingly, how passengers perceive the low-carbon expression is statistically significant, yet it has a negligible effect, while knowing the expressions is not statistically significant. Finally, those who voluntarily compensate for their CO_2_ emissions are also more willing to pay a higher price for a low-carbon fuel, and its effect size is medium.

**Table 3:** T-test and Cohen's d results for willingness to pay for a low-carbon fuel amongst passengers groups, where the level of significance was p = 0.05.

	Count	Mean	Variance	t	p-value	Cohen's d
**Age**				0.33	0.74	0.017
Under 55	507	11.51	284.61			
55 and older	1402	11.23	259.72			

**Gender**				-0.07	0.95	-0.0033
Female	632	11.19	238.90			
Male	1267	11.24	274.15			

**Education**				-3.99	6.93E-05	-0.20
No higher education	558	9.08	200.77			
Higher education	1310	12.35	291.41			

**Size of household**				0.29	0.77	0.0159
Maximum 2	1448	11.42	264.88			
More than 2	452	11.16	277.74			

**Frequency of travelling**				2.613	0.0090	0.1279
Maximum twice	1299	11.97	283.66			
More than twice	615	9.89	227.88			

**Spending on trips**				-6.54	7.86E-11	-0.299
Maximum 150	950	8.89	169.27			
More than 150	964	13.70	351.12			

**Choice of fuel**				3.98	7.34E-05	0.2612
Low-carbon fuel	756	12.89	317.99			
Non-low-carbon fuel	335	8.53	189.44			

**Fuel production method**				-5.24	1.84E-07	-0.3975
Fossil production	198	6.40	156.38			
Non-fossil production	1408	13.01	293.44			

**Knowledge of expressions**				-0.81	0.42	-0.074
Knows max half	1787	11.22	262.03			
Knows more than half	127	12.43	331.49			

**Emission mitigation solution**				4.28	1.94E-05	0.1958
Low-carbon fuel	958	12.89	294.60			
Other options	956	9.71	233.66			

**Voluntary compensation**				9.23	6.99E-20	0.4296
Would pay	777	15.38	339.01			
Would not pay	1138	8.51	198.15

**Table 4:** ANOVA and Eta squared results for willingness to pay for a low-carbon fuel amongst passengers groups, where the level of significance was p = 0.05.

	Count	Mean	Variance	Sum of squares		F	p-value	Eta squared
				Group	Within			
**Occupation**				1801.43	496608.65	1.12	0.35	0.0036
Student	42	8.18	130.84					
Employee	432	11.67	294.65					
Lower management	128	11.50	237.90					
Upper management	127	9.83	206.62					
Entrepreneur	179	13.59	345.25					
Unemployed	26	8.96	144.08					
Pensioner	931	11.31	261.23					

**Income**				2949.45	408100.5	5.41	0.0046	0.0072
Below 4000	831	10.75	238.82					
4000 to 10000	525	12.39	281. 87					
Above 10000	144	15.39	434. 82					

**Place of residence**				11621.45	498133.2	11.12	6.41E-09	0.0228
Denmark	282	12.08	289.85					
The Netherlands	241	16.30	411.41					
Norway	248	7.17	138.62					
The UK	851	10.40	216.96					
Other country	290	12.62	343.55					

**Environmental concern**				34523.72	475398.5	69.39	0	0.0677
Less concerned	324	4.33	95.70					
Concerned	743	9.38	183.95					
Very concerned	847	15.65	364.06					

**Impression of LC expression**				2081.75	507840.5	3.92	0.020	0.0041
Negative	150	13.39	408.75					
Neutral	722	10.06	253.56					
Positive	1042	11.86	253.72					

### 4.4 Discussion

It is clear from the results that passengers care about environmental issues and sustainability. They also seem to follow the news within the energy sector and the upcoming trends of new fuels and trends towards electrification. However, it is evident that only the most used terms will reach the general public and stay with them. It is mainly shown that respondents selected hydrogen as most suitable for RoPax operations, followed by electrification. It shows that they are aware that hydrogen is becoming one of the sustainable energy carriers and that the general media overwhelmingly writes about hydrogen. As shown by Rinscheid and Udris [73], the depth and tone of media coverage of energy policies and technology have a significant influence on the public's perceptions. However, it is also clear that the general public has no wide and deep knowledge about the current state of the direct applications of hydrogen. It might be possible that in the future, there will be significantly more battery-electric and hydrogen-fueled RoPax vessels in operation due to their economic and environmental benefits at shorter routes [74]. However, at the current technological level, these fuels are unavailable for long-distance shipping. When the passengers were provided with a short description of fuel production methods, they were more likely to select one, as compared to when only fuel types were provided without any context or description about them. Regarding fuel production methods, biofuels, when defined as first-generation biofuels, are not popular even though their perception was one of the most positive. This shows an agreement with the findings of Moula et al. [21], where the majority of respondents indicated that they would not buy biofuels if they were made from food crops. It can be said that passengers think similarly about fuels and their production as the authorities in the findings of Hansson et al. [34] and value environmental and social performance over economic and technical features.

Furthermore, many of the respondents admitted in the survey that they could not choose between fuels and described in the final comments that their knowledge does not cover these topics and that the survey was challenging to fill out. When the survey was conducted in person, it happened that passengers were thankful for the survey and for raising such an important issue that they have rarely thought about, even though they are using this service quite often. This is a rarely discussed topic, thus operators should take it upon themselves if they want their customers to be better informed. While the passengers have good intentions, there is evidently a knowledge gap. To overcome this gap, RoPax operators could educate their customers. As has been shown here and also elsewhere [75], [23], customers who are educated in general and especially on the given topic are more willing to bear additional costs for more sustainable products and services. This also agrees with the findings of Bertsch et al. [76], where acceptance of renewable energy sources was higher for those who possessed more knowledge on the topic. Perera et al. [77] has shown that young environmentalists have a learning curve in seeking information about more environmentally conscious products and companies. Furthermore, Giesler and Veresiu [75] also discusses green consumers who make responsible choices because they care about climate change which is adversely affected by their consumption and have realised that their decisions can have a positive influence. To reach those who are less informed, the vessel operators could provide information packages. Bertsch et al. [76] also recommends investments into energy education and transparent communication to increase the level of acceptance of renewable energy technologies. To create responsible consumers, easily reachable and understandable information materials must be generated. However, we recommend staying away from certain expressions unless they are clearly explained and instead using low-carbon fuel, sustainable marine fuel, green fuel and renewable fuel. Furthermore, there is a need to be careful with framing, as certain expressions or expressions without explanation induce negative feelings or, even worse, will be associated with greenwashing, which has appeared in the comments for voluntary CO_2_ compensation quite often. Building trust and transparency with the customers could enable their financial participation towards the energy transition of the marine sector.

## 5 Limitations and future work

In this study, the perceptions, awareness and WTP of RoPax passengers in the Northern European region were collected and analysed. The focus was two-fold: firstly, to measure RoPax passengers' knowledge and perception of fuel technologies, and secondly, to observe their willingness to pay either as an offset of their CO_2_ emissions or specifically for a low-carbon alternative fuel. To fulfil the first purpose, the survey was designed without any additional information about the fuels and their production technologies. Only minimal information was provided in order to make sure all the respondents understood some terms as it was intended. Lack of description and explanation of terms has led respondents to admit that they were unfamiliar with them and had to base their selections on information already in their possession. The lack of explanations was criticised by respondents in the free comment section as well, even though at the beginning of the survey, the purpose of the survey was described. Regarding the WTP part in future studies, it would be recommended to make a clear separation within the survey and inform the respondents about the benefits and drawbacks. Here, there was the possibility for the respondents to step back in the survey and change the already selected answers once the information had been provided. Therefore, we omitted to provide further information in the WTP part. Furthermore, our intention was to create an objective survey, and we can only hope that the respondents were not influenced by how the questions and answers were created.

Due to one of the data collection methods, the newsletter from one of the RoPax operators, a significant amount of responses originated from the countries where the operator provides their routes. Even though it was stated clearly at the beginning of the survey that it was from an academic institution and the answers were used solely for research purposes, several of the comments implied that the respondents believed that it was from the operator. We hope that there is no bias in the collected data and that respondents filled out the survey truthfully and according to the best of their knowledge, regardless of whom they believed collected the data. There was no response rejected from the submitted 1914 responses; all the responses can be deemed rational. Furthermore, as no information was collected to identify the respondents, the data collection cannot be replicated or reproduced, but only similar data collection can be carried out through the same channels with the same survey.

Regarding the WTP part, there are more sophisticated methods, just to name one, the contingent valuation method, that can measure and estimate the WTP more accurately [78]. Market data was nonexistent at the moment of writing, to our knowledge, there were no RoPax operators within the studied region that have collected this type of additional compensation. In fact, Viking Line has started to offer CO_2_ emission reduction options by purchasing biogas at less than 5 for their Turku-Åland-Stockholm route on the 21^st^ June 2023 [79]. Meanwhile, as WTP studies are theoretical studies and rarely follow through with actual payment, experimentation could be used as well.

In future studies, market data from the operators could be used to verify the results of WTP surveys, which is our intention in our follow-up study. Furthermore, the same study could be repeated in other regions, especially in the Southern European region, where similar RoPax vessels are operating. Different attitudes towards renewable energy technologies in different countries have been noted by Øystein Aas et al. [80] as well, which could be further analysed in future works. In the current study, the respondent's country of residence was a significant factor influencing the WTP, therefore, conducting the study in other regions would be beneficial. Finally, based on the collected data, a model estimating the WTP value could be generated and verified based on the market data.

## 6 Conclusions

Maritime transport is one of the hard-to-abate sectors of the transportation industry. There are ambitious goals and commitments to reach significant greenhouse gas emission reductions for the sector by 2050 within the European Union and beyond. These commitments are also in line with several of the United Nations' Sustainable Development Goals, such as “Clean water and sanitation”, “Good health and well-being”, “Affordable and clean energy”, and “Life below water”. The movement of passengers and products by sea transport is a significant mode of transport in the Northern region of Europe. In this research, we have investigated the willingness to pay of cruise-ferry passengers along with their general knowledge, awareness and perceptions of alternative fuels and other emission mitigation tools.

We collected close to 2000 answers from RoPax passengers who use this type of service. It is clear from our findings that well-informed passengers can make better decisions and are also willing to contribute financially to the energy transition. The average passenger has limited knowledge about the fuels and their applications within the sector, however, the most mainstream energy trends, such as electrification and the use of hydrogen, do reach them. They value environmental considerations of the fuel selected, and they also prefer production methods that generate lower emissions than fossil feedstocks. According to the passengers RoPax operators need to take responsibility for decreasing their emissions using low-carbon alternative fuels. Almost 80% of them are willing to pay increased prices for their fares if they know that the vessel utilises low-carbon fuels, and more than 40% are prepared to pay at least 5 more per one-way trip. To reach more of their passengers and increase financial participation, RoPax operators need to educate passengers as that leads to an improved understanding of the situation and makes them more involved in the green transition. In return, passengers are more likely to support these decisions financially when they are well articulated and argued for.

Even though our research mostly covers the Northern European region, participants were not involved from all the countries within this region, while some countries had a significantly higher representation. As the country of residence had a significant effect on WTP, it would be worthwhile to study other regions as well, such as the Southern European region, where similar RoPax vessels are in operation. Moreover, while the age group is in line with the average passengers' demographics, it would be beneficial to reach other age groups. Furthermore, as with other self-stated WTP studies, the payment only happens in theory and is not followed by actual payment. It would be intriguing to conduct a study where the theoretical statement is followed through by actual payment, and the disparity between the stated and actual WTP is studied.

## CRediT authorship contribution statement

**Judit Nyári:** Writing – review & editing, Writing – original draft, Visualization, Methodology, Investigation, Funding acquisition, Formal analysis, Conceptualization. **Árpád I. Toldy:** Writing – review & editing, Writing – original draft, Visualization, Methodology, Formal analysis. **Mika Järvinen:** Writing – review & editing, Writing – original draft, Supervision, Resources. **Annukka Santasalo-Aarnio:** Writing – review & editing, Writing – original draft, Supervision, Resources, Funding acquisition, Conceptualization.

## Declaration of Competing Interest

The authors declare that they have no known competing financial interests or personal relationships that could have appeared to influence the work reported in this paper.

## Data Availability

Data will be made available upon request.

## Appendix A Supplementary material

MMC Questionnaire for passengers. MMC
