# Supplementary material for: Awareness increases acceptance and willingness to pay for low-carbon fuels amongst marine passengers
Source: Heliyon. 2024 Jan 23;10(3):e24714. doi: 10.1016/j.heliyon.2024.e24714 (PMC10847608; doi:10.1016/j.heliyon.2024.e24714)
Supplement: MMC — Questionnaire for passengers. [file mmc1.pdf]

The purpose of this survey is to study passengers' views, habits and preferences about ferry ship travel in the Northern European region. The questionnaire specifically focuses on carbon dioxide emission and its reduction, especially on different types of fuel technologies.

There are no wrong or right answers to any of the questions, we are merely interested in your opinion. No previous knowledge is necessary, as one of the intentions of the questionnaire is to evaluate how familiar you are with certain expressions.

The answers are collected anonymously. The questions in this survey are created by doctoral students from Aalto University, Finland. The answers are collected for research purposes by Aalto University staff and will be shared with the participating ferry operator companies. The results of this survey can be used and published in scientific articles.

Answering this survey takes between 5-10 minutes.
Mandatory questions are marked with a star (*)

Part 1 - Background information

1. Which age group do you belong to?

   ◯ Under 18
   ◯ 18 - 34
   ◯ 35 - 54
   ◯ 55 - 74
   ◯ Over 75
   ◯ Do not wish to answer

2. What is your gender?*

   ◯ Female
   ◯ Male
   ◯ Other
   ◯ Do not wish to answer

3. What is your highest education level?*

   ◯ Compulsory education
   ◯ Vocational school
   ◯ High school
   ◯ Higher vocational
   ◯ Undergraduate
   ◯ Postgraduate
   ◯ Do not wish to answer

4. What is your occupation?*

◯ Student
◯ Employee
◯ Lower management
◯ Upper management
◯ Entrepreneur/Self-employed
◯ Unemployed
◯ Pensioner
◯ Do not wish to answer

5. What is the size of your household?*

◯ 1
◯ 2
◯ 3
◯ 4
◯ 5
◯ More than 5
◯ Do not wish to answer

6. What is the monthly net income of your household?*

◯ Below 200 €
◯ 201 - 500 €
◯ 501 - 1000 €
◯ 1001 - 1500 €
◯ 1501 - 2500 €
◯ 2501 - 4000 €
◯ 4001 - 6000 €
◯ 6001 - 8000 €
◯ 8001 - 10000 €
◯ 10001 - 15000 €
◯ More than 15000 €
◯ Do not wish to answer

7. Where do you live?*

◯ Denmark
◯ Estonia
◯ Finland
◯ France
◯ Germany
◯ Iceland
◯ Ireland
◯ Latvia
◯ Lithuania
◯ The Netherlands
◯ Norway
◯ Poland
◯ Russia
◯ Sweden
◯ United Kingdom
◯ Other, where:
◯ Do not wish to answer

Part 2 - Traveling customs and preferences

8. How often do you travel annually for leisure by ferries in Northern Europe? (see map)*

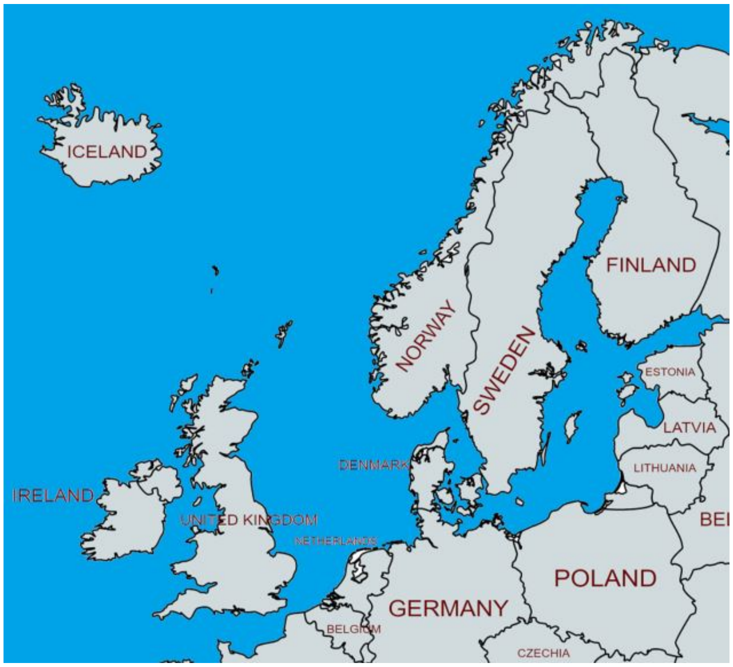

◯ Less than once per year
◯ 1 - 2 times
◯ 3 - 5 times
◯ More than 5 times

9. When you are choosing a ferry provider, what factors are the most important (Select max 3 options)*

☐ Timetable
☐ Route
☐ Environmental factors
☐ Location of ports and terminals
☐ Loyalty program
☐ Perceived quality of service
☐ Onboard entertainment and services
☐ Reputation of the ferry provider
☐ Services at destination offered by the ferry operator (Hotels, bus transportation, etc.)
☐ Price
☐ Other, what:

10. How much do you spend on average per trip per person (vehicle included, meals and other shopping not included)?*

◯ Below 20 €
◯ 20 - 50 €
◯ 51 - 100 €
◯ 101 - 150 €
◯ 151 - 200 €
◯ 201 - 300 €
◯ More than 300 €

Part 3 - Climate change mitigation and fuels

11. How concerned are you about environmental issues?*

    0 - not concerned at all   10 - very concerned

    Value:

12. What are the most serious environmental issue/s in your opinion? (Select max 3 options)*

    ☐ Overpopulation
    ☐ Water pollution
    ☐ Food security
    ☐ Deforestation
    ☐ Air pollution
    ☐ Acidification
    ☐ Global warming
    ☐ Loss of biodiversity
    ☐ Waste disposal
    ☐ Natural resource depletion
    ☐ Other, what:
    ☐ None of them

13. In your opinion, what are the most important characteristics a marine fuel needs to fulfil? Rank the listed characteristics in your preferred order.*

	1st	2nd	3rd	4th	5th	6th	7th
Availability	☐	☐	☐	☐	☐	☐	☐
Price	☐	☐	☐	☐	☐	☐	☐
Reliability	☐	☐	☐	☐	☐	☐	☐
Sustainable production option	☐	☐	☐	☐	☐	☐	☐
Low emission during utilisation	☐	☐	☐	☐	☐	☐	☐
Safety	☐	☐	☐	☐	☐	☐	☐
Non-harmful for the aquatic environment	☐	☐	☐	☐	☐	☐	☐

14. Which fuel do you think is the most suitable for ferry ships?*

◯ Low and very low sulfur oil (LSFO)
◯ Hydrogen
◯ Methanol
◯ Liquified natural gas (LNG)
◯ Ammonia
◯ Electricity
◯ Diesel
◯ Heavy fuel oil (HFO)
◯ Marine gas oil (MGO)
◯ Other, what:
◯ I cannot say

15. Which of the following production methods would you prefer to be used to produce the fuel for ferry ships?*

◯ Traditional fossil-based production (e.g. coal, natural gas, crude oil)
◯ Biomass-based production (produced directly from plants, e.g. corn, sugarcane)
◯ Renewable production (produced from wastes, residues or algae)
◯ Synthetic production (produced from renewable electricity)
◯ A combination of several methods (*Go to question 16*)
◯ No preference

16. You selected that you would like to utilize a combination of several fuel production methods. Please select which methods you would prefer (max 2).*

☐ Traditional fossil-based production (e.g. coal, natural gas, crude oil)
☐ Biomass-based production (produced directly from plants, e.g. corn, sugarcane)
☐ Renewable production (produced from wastes, residues or algae)
☐ Synthetic production (produced from renewable electricity)

17. Which of the following fuel technologies have you heard before? Mark all those you are familiar with.*

☐ Emission-to-Liquids (EtL)
☐ Biofuel
☐ Power-to-Liquids (PtL)
☐ Sustainable marine fuel
☐ Synthetic fuel
☐ Alternative marine fuel
☐ Green fuel
☐ Powerfuel

☐ Low-carbon fuel
☐ Power-to-X (PtX, P2X)
☐ Electrofuel
☐ Renewable fuel
☐ Fossil fuel
☐ E-fuel
☐ None of them

18. What kind of view or impression do you have on the following fuel technologies?
It does not matter if you are not familiar with the expression. We would like to know what kind of impression you have about the expression when you hear or see it written.

	Negative	Slightly negative	Neutral	Slightly positive	Positive
Emission-to-Liquids (EtL)	☐	☐	☐	☐	☐
Biofuel	☐	☐	☐	☐	☐
Power-to-Liquids (PtL)	☐	☐	☐	☐	☐
Sustainable marine fuel	☐	☐	☐	☐	☐
Synthetic fuel	☐	☐	☐	☐	☐
Alternative marine fuel	☐	☐	☐	☐	☐
Green fuel	☐	☐	☐	☐	☐
Powerfuel	☐	☐	☐	☐	☐
Low-carbon fuel	☐	☐	☐	☐	☐
Power-to-X (PtX, P2X)	☐	☐	☐	☐	☐
Electrofuel	☐	☐	☐	☐	☐
Renewable fuel	☐	☐	☐	☐	☐
Fossil fuel	☐	☐	☐	☐	☐
E-fuel	☐	☐	☐	☐	☐

19. Who do you think should be mainly responsible for decreasing the carbon dioxide emissions in marine passenger transportation?*

◯ Passengers
◯ Ferry operators
◯ Fuel suppliers
◯ National governments
◯ The European Union
◯ International non-governmental organisations such as the International Maritime Organization
◯ None of the above
◯ Other, who:

20. If you had to choose, how would you reduce greenhouse gas emissions from marine passenger transportation?*

◯ By reducing marine passenger traffic
◯ By using low-carbon fuel alternatives
◯ By increasing the taxes on carbon dioxide emissions
◯ By improvements in engines
◯ With better weather forecasting and navigational technologies
◯ None of the above
◯ Other, what:

Part 4 - Willingness-to-pay

21. If there would be a voluntary option to compensate for the carbon dioxide emission of your trip, would you purchase such an option?*

◯ No (*Go to question 22*) ◯ Yes (*Go to question 23*)

22. You indicated that you would not compensate for your carbon dioxide emission voluntarily. What is the main reason for this?*

◯ I do not believe in climate change
◯ Climate change does not affect me
◯ I prefer to spend my money on other things
◯ My income is too low
◯ I do not believe that the compensation will have any real impact
◯ I do not believe that the collected compensation is spent on carbon dioxide mitigation
◯ Other, what:

Go to question 27.

23. How would you determine the amount of carbon dioxide compensation?*

◯ Fixed amount regardless of the trip (*Go to question 24*)
◯ Based on the distance of the trip (*Go to question 25*)

24. How much would you be willing to pay per one-way per person for carbon dioxide emission compensation?*

◯ Max 0.5 €
◯ Max 1 €
◯ Max 2.5 €
◯ Max 5 €
◯ Max 10 €
◯ Max 25 €
◯ Max 50 €
◯ More than 50 €

Go to question 26.

25. How much would you be willing to pay per 100 kilometres per person for carbon dioxide emission compensation?*

◯ Max 0.05 €
◯ Max 0.1 €
◯ Max 0.25 €
◯ Max 0.5 €
◯ Max 1 €
◯ Max 2.5 €
◯ Max 5 €
◯ More than 5 €

26. How should the ferry operator spend the collected compensation?*

◯ Send it to non-profit organisations
◯ Invest it in carbon dioxide mitigation equipment
◯ Buy low-carbon fuel
◯ Use it as a general budget as the ferry provider sees fit
◯ Return it to the local government as a tax
◯ It does not matter to me
◯ Other, what:

27. How much more would you be willing to pay for a ticket per person per one-way if the fuel used would be a low-carbon alternative?*
A low-carbon fuel means that it either emits a lower amount of carbon dioxide during its utilisation or during its production than the average standard fuel.

◯ Max 0.5 €
◯ Max 1 €
◯ Max 2.5 €
◯ Max 5 €
◯ Max 10 €
◯ Max 25 €
◯ Max 50 €
◯ More than 50 €
◯ Would not pay more (*Go to question 28*)

28. You indicated that you are not willing to pay more for a low-carbon fuel. What is the main reason for this?*

◯ I do not believe in climate change
◯ Climate change does not affect me
◯ I prefer to spend my money on other things
◯ My income is too low
◯ I do not believe that the climate surcharge on fuels will have any real impact
◯ I believe that the currently used fuel is suitable
◯ The current carbon tax on fuels is enough
◯ Other, what:

29. If you have any comments or thoughts regarding the survey, feel free to express them here. Remember to submit your answers when you are ready.

....
....
....
....
....
....
....
....
....
....
....
....
....
....
....
....

Thank you four your participation!
